# Legumain – Outra Peça no Complexo Quebra-Cabeça da Formação e Vulnerabilidade da Placa Aterosclerótica

**DOI:** 10.36660/abc.20230743

**Published:** 2023-12-07

**Authors:** Ana Teresa Timóteo

**Affiliations:** 1 Serviço de Cardiologia Hospital Santa Marta Centro Hospitalar Universitário Lisboa Central Portugal Serviço de Cardiologia , Hospital Santa Marta - Centro Hospitalar Universitário Lisboa Central , Lisboa – Portugal; 2 Faculdade de Medicina NOVA Faculdade de Ciências Médicas Universidade Nova Lisboa Portugal Faculdade de Medicina NOVA - Faculdade de Ciências Médicas - Universidade Nova Lisboa , Lisboa – Portugal

**Keywords:** Cisteína Proteases, Placa Aterosclerótica, Doença Arterial Coronariana, Angina Instável, Ultrassonografia de Intervenção/métodos, Biomarcadores

O processo de formação de placas ateroscleróticas nas paredes arteriais é um processo muito complexo. O desenvolvimento da placa ocorre devido a uma interação entre células endoteliais, células musculares lisas e células imunológicas. ^1^ Há aumento da permeabilidade endotelial e ativação de moléculas sinalizadoras, incluindo mediadores inflamatórios e moléculas de adesão celular. ^1^ Além disso, as lipoproteínas circulantes (particularmente as lipoproteínas de baixa densidade – LDL) podem atravessar a camada endotelial e depositar-se no espaço íntimo, onde sofrem alterações oxidativas (LDLox), aumentando a resposta inflamatória local. ^1^ Além disso, os monócitos amadurecem em macrófagos, sendo recrutados através de quimiocinas atrativas, e enquanto tentam eliminar o LDLox através de fagocitose, são convertidos em células espumosas e, por fim, sofrem necrose. ^1^ Além disso, as células musculares lisas vasculares (CMLVs) proliferam e migram da camada média para a íntima, levando à hiperplasia intimal, e essas células se organizam e depositam matriz extracelular, formando uma capa fibrosa. ^1^ A erosão ou ruptura da capa fibrosa levará à oclusão trombótica ou embolia distal, resultando em isquemia. ^1^

Neste processo, as metaloproteinases de matriz (MMPs) desempenham um papel importante. As MMPs são uma família de endoproteases dependentes de zinco que são secretadas por células endoteliais, CMLVs, fibroblastos, osteoblastos, macrófagos, neutrófilos e linfócitos. ^2^ Durante o desenvolvimento estável da placa aterosclerótica, a MMP-2 cliva a sintase do óxido nítrico endógeno, causando disfunção endotelial e facilitando a infiltração de LDL na íntima. ^2^ Então, a MMP-2 expressa por plaquetas ativadas promove a transmigração de monócitos para a íntima. ^2^ A MMP-2 também facilita a migração de CMLVs induzidas por LDLox para a íntima, formando a capa fibrosa. ^2^ Portanto, pode induzir a migração e proliferação de CMLVs, aumentando a estabilidade da placa. No entanto, os níveis de MMP-2 são mais elevados em placas ateroscleróticas instáveis do que em estáveis, indicando que a MMP-2 desempenha um papel na vulnerabilidade da placa e na propensão à ruptura. ^2^ Participam da degradação da matriz extracelular (MEC), o que causará adelgaçamento da capa fibrosa da placa, tornando-a mais propensa à ruptura. ^2^

Legumain é uma cisteína protease endolisossomal, também conhecida como asparaginil endopeptidase, que está localizada principalmente nos lisossomos, embora também seja encontrada extracelularmente como uma proteína secretada. ^3 - 6^ É altamente expressa em diversos órgãos, bem como na patogênese de diversas doenças malignas e não malignas, como doenças cardiovasculares e cerebrovasculares, fibrose, envelhecimento e senescência, doenças neurodegenerativas e câncer. ^3 , 5^ Possui atividade proteolítica dependente do pH (portanto regulada pelo meio ambiente), mediando a ativação, o processamento ou a degradação de diferentes substratos. ^3^ Pode ativar a MMP-2 e clivar e degradar a fibronectina, que controla a remodelação da MEC. ^3^ Também pode regular a função das células imunológicas e tem um papel vital na apresentação de antígenos durante a resposta inflamatória. Além disso, tem também função de ligase não proteolítica. ^3^ Estudos anteriores sugeriram um papel da legumain no desenvolvimento e ruptura da placa aterosclerótica devido à influência nas MMPs. Na verdade, foi descrito que a legumain é regulada positivamente em placas carotídeas instáveis e em pacientes com acidente vascular cerebral, sugerindo um papel potencial na vulnerabilidade e instabilidade da placa. ^6^ No entanto, um efeito protetor também foi descrito. Promove uma mudança em direção a um fenótipo anti-inflamatório de macrófagos e promove depuração e degradação de cardiomiócitos apoptóticos após infarto do miocárdio. ^3^

Nesta edição da revista, Deng et al., ^7^ estudaram a associação da legumain com características da placa aterosclerótica coronariana ^.^ Eles incluíram 81 pacientes com doença arterial coronariana (DAC) e um grupo controle. A ultrassonografia intravascular foi utilizada para avaliar as características das placas ateroscleróticas coronarianas. Os níveis de legumain foram significativamente maiores em pacientes com DAC, principalmente no grupo instável. Pacientes instáveis apresentaram maior área de remodelação e índice de excentricidade, ambos significativa e positivamente correlacionados com os níveis de legumain. Os níveis de legumain foram preditores independentes de angina instável e mostraram boa precisão preditiva por uma área sob a curva ROC de 0,789. Um ponto de corte de 21,7 ng/mL apresentou sensibilidade de 65% e especificidade de 92% para angina instável. Portanto, os autores concluem que este biomarcador pode ser útil na detecção de lesões coronárias instáveis.

Esta pesquisa é relevante porque contribui para o conhecimento conceitual dos processos por trás da formação e vulnerabilidade da placa aterosclerótica, somando-se às informações já disponíveis. Se a ultrassonografia intravascular com histologia virtual tivesse sido utilizada, mais informações poderiam ter sido coletadas sobre a composição da placa. No entanto, não pode ser utilizado na prática clínica porque a medição da legumain não está atualmente disponível em laboratórios clínicos. No entanto, os resultados atuais abrem uma porta para futuras pesquisas sobre alvos de tratamento relacionados à vulnerabilidade da legumain e da placa aterosclerótica. Já foi demonstrado anteriormente que medicamentos como as estatinas são capazes de inibir a atividade da legumain. ^3^ Além disso, os inibidores da bomba de prótons (IBP) também podem inibir diretamente a legumain por ligação à cisteína no sítio ativo. ^3^ No entanto, os mecanismos exatos e as consequências da inibição da legumain pelas estatinas ou IBPs continuam por elucidar, e são necessárias mais pesquisas nesta área.
