# Supplementary material for: Legumain – Outra Peça no Complexo Quebra-Cabeça da Formação e Vulnerabilidade da Placa Aterosclerótica
Source: Arq Bras Cardiol. 2023 Dec 7;120(11):e20230743. [Article in Portuguese] doi: 10.36660/abc.20230743 (PMC10802184; doi:10.36660/abc.20230743)
Supplement: Translation

# Translation: Legumain – Another Piece in the Complex Puzzle of Atherosclerotic Plaque Formation and Vulnerability

**Authors:** Ana Teresa Timóteo

**Affiliations:** 1 Portugal Serviço de Cardiologia, Hospital Santa Marta - Centro Hospitalar Universitário Lisboa Central, Lisboa – Portugal; 2 Portugal Faculdade de Medicina NOVA - Faculdade de Ciências Médicas - Universidade Nova Lisboa, Lisboa – Portugal

**Keywords:** Cysteine Proteases, Plaque,Atherosclerosis, Coronary Artery Disease, Angina, Unstable, Ultrasonography, Interventional/methods, Biomarkers

The atherosclerotic plaque formation process in the arterial walls is a very complex process. Plaque development occurs due to an interplay between endothelial cells, smooth muscle cells, and immune cells. ^1^ There is an increase in endothelial permeability and activation of signaling molecules, including inflammatory mediators and cell adhesion molecules. ^1^ Moreover, circulating lipoproteins (particularly low-density lipoproteins – LDL) can cross the endothelial layer and be deposited in the intimal space, where they undergo oxidative changes (oxLDL), increasing the local inflammatory response. ^1^ Furthermore, monocytes mature into macrophages, being recruited via attractant chemokines, and while they attempt to clear oxLDL via phagocytosis, they are converted into foam cells and ultimately undergo necrosis. ^1^ In addition, vascular smooth muscle cells (VSMCs) proliferate and migrate from the media to the intima, leading to intimal hyperplasia, and these cells organize and deposit extracellular matrix, forming a fibrous cap. ^1^ Erosion or rupture of the fibrous cap will lead to thrombotic occlusion or distal embolism, resulting in ischemia. ^1^

In this process, matrix metalloproteinases (MMPs) play an important role. MMPs are a family of zinc-dependent endoproteases that are secreted by endothelial cells, VSMCs, fibroblasts, osteoblasts, macrophages, neutrophils, and lymphocytes. ^2^ During stable atherosclerotic plaque development, MMP-2 cleaves the endogenous nitric oxide synthase, causing endothelial dysfunction and facilitating infiltration of LDL into the intima. ^2^ Then, MMP-2 expressed by activated platelets promotes monocyte transmigration into the intima ^2^ . MMP-2 also facilitates oxLDL-induced VSMCs migration to the intima, forming the fibrous cap. ^2^ Therefore, it can induce migration and proliferation of VSMCs, enhancing plaque stability. However, MMP-2 levels are higher in unstable than in stable atherosclerotic plaque, indicating that MMP-2 plays a role in plaque vulnerability and propensity to rupture. ^2^ They participate in the degradation of the extracellular matrix (ECM), which will cause thinning of the plaque fibrous cap, becoming more prone to rupture. ^2^

Legumain is an endolysosomal cysteine protease, also known as asparaginyl endopeptidase, that is mainly located in the lysosomes, although it is also found extracellularly as a secreted protein. ^3 - 6^ It is highly expressed in several organs, as well as in the pathogenesis of several malignant and non-malignant diseases, such as cardiovascular and cerebrovascular diseases, fibrosis, aging and senescence, neurodegenerative diseases, and cancer. ^3 , 5^ It has a proteolytic activity that is pH-dependent (therefore regulated by the environment), mediating either the activation, processing, or degradation of different substrates. ^3^ It can activate MMP-2 and cleavage and degrade fibronectin, which controls ECM remodeling. ^3^ It can also regulate immune cell function and has a vital role in antigen presentation during the inflammatory response. In addition, it has also a nonproteolytic ligase function. ^3^ Previous studies suggested a role for legumain in atherosclerotic plaque development and rupture due to the influence in MMPs. In fact, it has been described that legumin is upregulated in unstable carotid plaques and patients with stroke, suggesting a potential role in plaque vulnerability and instability. ^6^ Nevertheless, a protective effect has also been described. It promotes a shift towards an anti-inflammatory macrophage phenotype and promotes clearance and degradation of apoptotic cardiomyocytes after myocardial infarction. ^3^

In this issue of the journal, Deng et al. ^7^ studied the association of legumain with coronary atherosclerotic plaque characteristics. They included 81 patients with coronary artery disease (CAD) and a control group. Intravascular ultrasound was used to evaluate the characteristics of coronary atherosclerotic plaques. Legumin levels were significantly higher in patients with CAD, particularly in the unstable group. Unstable patients had a higher remodeling area and eccentricity index, both significantly and positively correlated with legumin levels. Legumain levels were independent predictors of unstable angina and showed good predictive accuracy by an area under the ROC curve of 0.789. A cut-off of 21.7 ng/mL had a sensitivity of 65% and specificity of 92% for unstable angina. Therefore, the authors conclude that this biomarker can be useful in the detection of unstable coronary lesions.

This research is relevant because it contributes to the conceptual knowledge of the processes behind atherosclerotic plaque formation and vulnerability, adding to information already available. If virtual histology-intravascular ultrasound had been used, more information could have been gathered regarding plaque composition. However, it cannot be used in clinical practice because legumain measurement is not currently available in clinical labs. Nevertheless, the current results open a door for further research in treatment targets related to legumain and plaque vulnerability. It has been previously shown that drugs such as statins are able to inhibit legumain activity. ^3^ In addition, proton pump inhibitors (PPI) can also directly inhibit legumain by binding to the cysteine in the active site. ^3^ However, the exact mechanisms and consequences of legumain inhibition by statins or PPIs remain to be elucidated, and further research in this area is warranted.
